# Critical Components for Spontaneous Activity and Rhythm Generation in Spinal Cord Circuits in Culture

**DOI:** 10.3389/fncel.2020.00081

**Published:** 2020-04-28

**Authors:** Samuel Buntschu, Anne Tscherter, Martina Heidemann, Jürg Streit

**Affiliations:** Department of Physiology, University of Bern, Bern, Switzerland

**Keywords:** rhythm generation, intrinsic spiking, TRP channels, multielectrode arrays, central pattern generator, I_CAN_, I_NaP_

## Abstract

Neuronal excitability contributes to rhythm generation in central pattern generating networks (CPGs). In spinal cord CPGs, such intrinsic excitability partly relies on persistent sodium currents (I_NaP_), whereas respiratory CPGs additionally depend on calcium-activated cation currents (I_CAN_). Here, we investigated the contributions of I_NaP_ and I_CAN_ to spontaneous rhythm generation in neuronal networks of the spinal cord and whether they mainly involve Hb9 neurons. We used cultures of ventral and transverse slices from the E13–14 embryonic rodent lumbar spinal cord on multielectrode arrays (MEAs). All cultures showed spontaneous bursts of network activity. Blocking synaptic excitation with the AMPA receptor antagonist CNQX suppressed spontaneous network bursts and left asynchronous intrinsic activity at about 30% of the electrodes. Such intrinsic activity was completely blocked at all electrodes by both the I_NaP_ blocker riluzole as well as by the I_CAN_ blocker flufenamic acid (FFA) and the more specific TRPM4 channel antagonist 9-phenanthrol. All three antagonists also suppressed spontaneous bursting completely and strongly reduced stimulus-evoked bursts. Also, FFA reduced repetitive spiking that was induced in single neurons by injection of depolarizing current pulses to few spikes. Other antagonists of unspecific cation currents or calcium currents had no suppressing effects on either intrinsic activity (gadolinium chloride) or spontaneous bursting (the TRPC channel antagonists clemizole and ML204 and the T channel antagonist TTA-P2). Combined patch-clamp and MEA recordings showed that Hb9 interneurons were activated by network bursts but could not initiate them. Together these findings suggest that both I_NaP_ through Na^+^-channels and I_CAN_ through putative TRPM4 channels contribute to spontaneous intrinsic and repetitive spiking in spinal cord neurons and thereby to the generation of network bursts.

## Introduction

Central pattern generator networks (CPGs) provide rhythmic output to muscles to support repetitive movements used in locomotion or breathing (Feldman et al., 2013; Kiehn, 2016). The mechanisms involved in such rhythm generation are still not fully understood. Especially, it is not clear, which types of interneurons contribute to rhythm generation, which circuits they form and which ion channels and receptors contribute to their intrinsic excitability. In the lumbar spinal cord, several types of genetically identified interneurons (Hb9, Shox 2 and V2a) have been proposed to participate in CPG networks (Ziskind-Conhaim et al., 2010; Dougherty et al., 2013; Ljunggren et al., 2014). For Hb9 interneurons, however, a key role for rhythm generation is still under debate (Caldeira et al., 2017).

In the mammalian spinal cord, the persistent sodium current I_NaP_ has been proposed to be involved in rhythm generation. This current is activated at sub-threshold potentials around −60 mV and probably represents a special state of the voltage-dependent Na^+^ channel (Urbani and Belluzzi, 2000). It contributes to intrinsic spiking of neurons and rhythm generation in organotypic and dissociated cultures of the spinal cord (Darbon et al., 2004; Yvon et al., 2007; Czarnecki et al., 2009) as well as in the neonatal rat spinal cord (Tazerart et al., 2007, 2008; Ziskind-Conhaim et al., 2008).

In respiratory circuits of the brainstem, two types of ion channels are proposed and debated to contribute to intrinsic firing of pacemaker neurons and thus participate in rhythm and pattern generation (Del Negro et al., 2005, 2018; Pace et al., 2007a,b; Koizumi et al., 2018; Picardo et al., 2019). In the first type of neurons, intrinsic spiking is based on I_NaP_, in the second type on a cation current that is activated by Ca^2+^ (I_CAN_). I_CAN_ is believed to be mediated by TRPM and/or TRPC channels (Ben-Mabrouk and Tryba, 2010; Mrejeru et al., 2011). It has been shown to underlie sustained depolarization, persistent activity and rhythm generation in a subset of neurons in the pre-Bötzinger complex (Pace et al., 2007b; Del Negro et al., 2010) and other neuronal circuits (Schiller, 2004; Mrejeru et al., 2011). In the spinal cord there is so far only some evidence for the role of I_CAN_ in intrinsic spiking of dorsal horn neurons but not in motor rhythm generation in the ventral spinal cord (Wang et al., 2006; Li and Baccei, 2011).

Rhythm generation is also a prominent feature in isolated neuronal networks in culture. In organotypic cultures of transverse slices of embryonic rat spinal cord, rhythms consisting of bursting activity with intraburst oscillations have been described previously (Ballerini et al., 1999; Tscherter et al., 2001). We demonstrated that this type of spontaneous rhythmic activity is based on intrinsic firing in a subset of neurons and on glutamatergic excitatory and recurrent GABA-ergic inhibitory connections in the network (Czarnecki et al., 2008). Also, we showed that intrinsic firing depends on I_NaP_ and hyperpolarization-activated cation currents I_h_ (Darbon et al., 2004).

In the present article, we show that similar rhythms are produced in ventral circuits of the rat spinal cord in longitudinal slices cultured on multielectrode arrays (MEAs) as previously described in cultures from transverse slices. We then investigated the relative contributions of I_NaP_ and I_CAN_ to intrinsic spiking and rhythm generation and we searched for a putative role of HB9 interneurons in the generation of bursting activity.

## Materials and Methods

### Culture Preparation

Cultures were obtained from spinal cords of either 14 days old rat embryos (E14) from Wistar rats purchased from Janvier (Le Genest St Isle, France) or of 13-day old embryos from Hlxb9-GFP mice [B6.CG-TG(Hlxb9-GFP)1Tmj/J] that express a green fluorescent protein (GFP) under the Hlxb9 promoter (obtained from Jackson Laboratory). The embryos were delivered by cesarean section from deeply anesthetized pregnant animals (after an intramuscular injection of 0.4 ml pentobarbital, Streuli Pharma SA, Switzerland), followed by an additional intraperitoneal application of pentobarbital. Deep anesthesia was confirmed before the section using the withdrawal reflex of the hind paw. This procedure guaranteed minimal suffering of animals (grade 0). The number of animals used to prepare the cultures was kept to a minimum. Animal care was under guidelines approved by Swiss local authorities (Amt für Landwirtschaft und Natur des Kantons Bern, Veterinärdienst, Sekretariat Tierversuche, approval Nr. BE 52/11 and BE 35/14). These guidelines are in agreement with the European Community Directive 86/609/EEC. After decapitation of the embryos, the lumbar parts of the backs were cut out and isolated from their limbs and viscera. Then they were cut into four to five 225 μm thick frontal slices with a tissue chopper and kept at 4°C in the slicing medium (Dulbecco’s Modified Eagle’s Medium with Glutamax, 25 mM Hepes and Antibiotics). Custom-made MEAs (external size 21 × 21 mm, Qwane Biosystems, Lausanne, Switzerland) were coated for 1 h with diluted (1:50) Matrigel^®^ (Falcon/Biocoat, Becton Dickinson AG, Switzerland). After dissecting the spinal cord slices from the surrounding tissue the two most ventral of them were fixed on top of each MEA by using reconstituted chicken plasma coagulated by thrombin (both Sigma–Aldrich, Switzerland). In addition to the cultures of longitudinal slices from rat spinal cord, we also prepared cultures of transverse slices from mouse and rat spinal cord as previously described (Tscherter et al., 2001). The cultures were maintained in sterile plastic tubes containing 3 ml of nutrient medium and incubated in roller drums rotating at 1 r.p.m in a 5% CO_2_-containing atmosphere at 36.5°C (Streit et al., 2001). The medium was composed of 79% Dulbecco’s modified Eagle’s medium with Glutamax, 10% horse serum (both Gibco BRL, Switzerland), 10% H_2_O and 5 ng/ml 2.5S nerve growth factor (Sigma–Aldrich, Switzerland). Half of the medium was replaced once to twice per week.

### MEA Recording and Analysis

**Figure 1:**
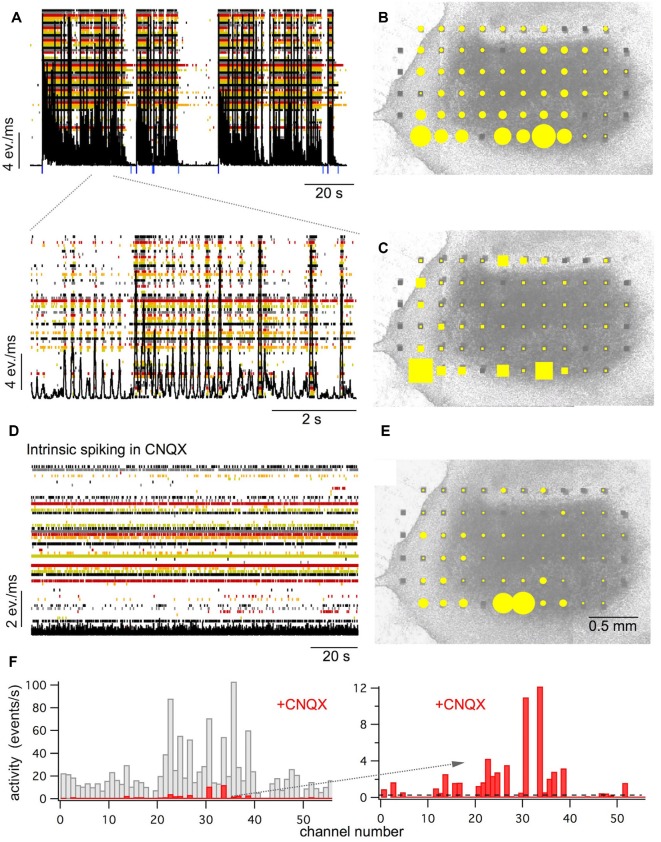
Spontaneous activity in longitudinal spinal cord slice cultures. **(A)** Raster plot of the activity recorded by 61 electrodes (in color) superimposed by the network activity plot (black). Spontaneous activity is composed of network bursts and intraburst oscillations (see lower extract with higher time resolution). Blue marks show the starts and stops of the detected bursts. **(B)** The activity distribution plot (yellow dots) superimposed on a picture of the culture at 30 days *in vitro*. The size of the yellow dots represents the relative amount of multiple unit activity recorded at the electrode within 10 min. **(C)** Sites where bursts start with high probability (burst sources: yellow square size shows the percentage of bursts starting at this electrode) superimposed on a picture of the culture. **(D)** Raster and network activity plot after blockade of the fast glutamatergic synaptic transmission with the AMPA receptor antagonist CNQX (10 μM). Note that network bursts are suppressed and irregular low-level activity (intrinsic spiking) at about 30–40% of the electrodes appears. **(E)** The activity distribution plot for intrinsic activity (yellow dots) recorded after blockade of the fast glutamatergic synaptic transmission with CNQX superimposed on a picture of the culture. **(F)** Channel activity histograms for bursting activity as shown under **(A,B**; gray) and for intrinsic activity in the presence of CNQX as shown under **(D,E**; red). Extract on the right side shows intrinsic activity at higher resolution. The dashed line shows the detection threshold for intrinsic activity set at 0.1 events/s (see also Figure 2). All recordings shown under **(A–E)** are from the same slice.

MEAs consisted of 68 platinum-plated electrodes laid out in the form of a rectangle. The electrodes measured 40 μm × 40 μm and were spaced 200 μm apart (center to center, e.g., Figure 1A). Recordings were made from cultures of 3–10 weeks *in vitro* age. An MEA with culture was mounted in a recording chamber on the stage of an upright microscope (Olympus BX 45, Tokyo, Japan) of a patch-clamp setup that was equipped with fluorescence microscopy settings to allow for the visualization of GFP-expressing neurons. The medium was replaced by an extracellular solution containing (in mM): NaCl, 145; KCl, 4; MgCl_2_, 1; CaCl_2_, 2, HEPES, 5; Na-pyruvate, 2; glucose, 5; pH 7.4. Recordings were made 5 min after the solution change in the absence of continuous superfusion with a solution change every 10–15 min. All recordings were made at room temperature (RT; 24 ± 4°C). Under these conditions, the cultures showed spontaneous network bursting activity that usually originated from all over the slices and remained stable for several hours.

Electrodes that were covered by the spinal cord slice (usually 40–60) were selected by eye and their recordings digitized at 6 kHz, visualized and stored on the hard disc using custom-made virtual instruments within Labview (National Instruments, Switzerland), as described previously (Streit et al., 2001). Detection of the extracellularly recorded action potentials and further analysis were done offline with the software package IGOR (WaveMetrics, Lake Oswego, OR, USA) as described previously (Tscherter et al., 2001). The detected signals were fast voltage transients (<4 ms), which correspond to single action potentials in neurons (somata or axons, single-unit activity). These transients often appeared in clusters (multi-unit activity) originating from closely timed action potentials of several neurons recorded by one electrode. When they appeared at more than 250 Hz (= upper limit of temporal resolution of the detector), they could not be separated from each other and therefore such activity was set by definition to 333 Hz (Tscherter et al., 2001). No attempt was made to sort the spikes recorded by one electrode. The selectivity of event detection for spiking activity was assessed using recordings obtained in the presence of tetrodotoxin (TTX, 1.5 μM) as a zero reference.

**Figure 2:**
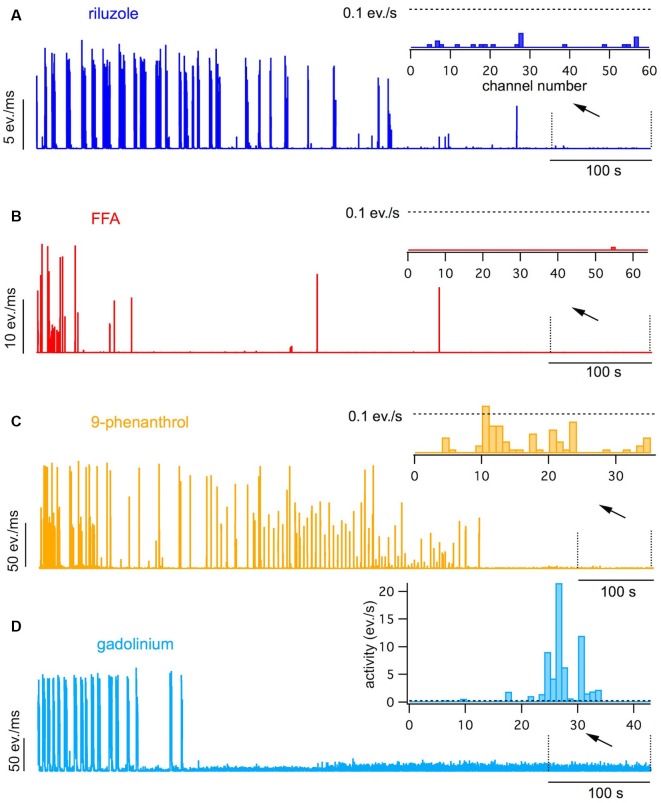
Effects of 20 μM riluzole **(A)**, 100 μM flufenamic acid (FFA; **B**), 100 μM 9-phenanthrol **(C)** and 30 μM gadolinium chloride **(D)** on spontaneous bursting and intrinsic activity. Network activity plots obtained during exposure to the antagonists following a pre-exposition to the antagonist for 15–20 min are shown. Insets show the channel activity histograms obtained in the last 100 s of the exposure. Dotted lines show the detection threshold for intrinsic activity set a 0.1 events/s. Note that all antagonists completely blocked spontaneous bursting, but only riluzole, FFA and 9-phenanthrol completely suppressed intrinsic activity.

The processed data were displayed as event raster plots, network activity plots, and activity distribution plots (see Figure 1). Event raster plots show the time markers of the detected activity of each selected electrode (= channel). Network activity plots show the total activity of all selected channels summed within a sliding window of 10 ms, shifted by 1 ms steps. Activity distribution plots show filled circles whose diameters are proportional to the total activity of the electrodes projected on a picture of the slice culture. Total activity is measured as the mean of the activity per second detected by each electrode during the whole recording period (usually 10 min). From the TTX reference, the selectivity threshold for each channel was set at 0.1 events/s. Channels with an activity above the threshold were defined as active (see Figure 2).

We defined clusters of activity that appear at several electrodes as bursts. Bursts were detected by setting a threshold at 50% of maximum activity in network activity plots and by defining the burst start as the time of the first event within this burst after a minimum silent period of 5 ms and the burst end as the time of the last event before a minimum silent period of 200 ms. The frequency of oscillations within the bursts were determined by counting the peaks of network activity plots within the first 1–2 s of a burst. For each burst, we identified the start point as the channel that first showed activity. Burst sources were defined as channels where bursts started more often than expected from the quotient of the number of bursts vs. the number of active channels.

### Electrical Stimulation with MEA Electrodes

Electrical stimulation was done with MEA electrodes using monopolar biphasic stimuli with a duration of 0.1 ms and an amplitude of 1–2 V that was delivered from a custom-made stimulator. The stimuli were applied to one electrode. The evoked events were recorded at all other MEA electrodes and quantified as network activity in a time window following the stimuli.

### Whole-Cell Patch-Clamp Recording and Analysis

Intracellular voltage measurements were obtained from individual neurons in the slice using the whole-cell patch-clamp technique with an Axopatch 200 amplifier (Molecular Devices Inc., Sunnyvale, CA, USA). For patch-clamp experiments, we used continuous superfusion (1.5 ml/min) at RT. The extracellular solution was the same as for MEA experiments. The patch pipettes were filled with a solution containing (in mM): K-gluconate 120; KCl 10; HEPES, 10; Mg-ATP, 4; Na_2_-GTP, 0.3; Na_2_-phosphocreatine, 10; pH 7.3 (with KOH). They were connected to the amplifier using a chloride silver wire. The electrodes had a resistance of 3–4 MΩ. No series resistance compensation was applied. Native resting membrane potentials were in the range of –45 to –75 mV. A junction potential of 15 mV was systematically corrected. The recordings were digitized at 6–10 kHz, visualized and stored on a computer using pClamp software (Molecular Devices Inc.). The signals were analyzed offline using custom-made programs in IGOR (WaveMetrics, Lake Oswego, OR, USA) and Clampfit software (Molecular Devices Inc).

### Immunohistochemistry

Cultures were fixed with 4% paraformaldehyde in phosphate-buffered saline (PBS, 0.1 M, pH 7.4) for 1–2 h at 4°C and then rinsed three times in PBS (0.1 M, pH 7.4) and stored at 4°C until used. Cultures were incubated in 2% chicken or 2% donkey serum, 0.5% bovine serum albumin (BSA) and 0.5% Triton X-100 in PBS (0.1 M, pH 7.4; blocking solution) for 1 h at RT and subsequently incubated in a humid chamber overnight at 4°C with goat polyclonal antibody to choline acetyltransferase (ChAT; AB144P; Chemicon, 1:100), diluted and mixed in PBS (0.1 M, pH 7.4), Triton X-100 (0.5%), BSA (0.5%). Cultures were then rinsed three times in PBS (0.1 M, pH 7.4) Triton X-100 (0.5%) and incubated with Alexa fluor 488 conjugated chicken polyclonal anti-goat antibody (A-21467; Invitrogen, 1:200) for 2 h at RT. This component was diluted and mixed in PBS (0.1 M, pH 7.4), Triton X-100 (0.5%).

Finally, samples were rinsed three times in PBS (0.1 M, pH 7.4) and then mounted with mowiol (4–88 Fluka from Sigma–Aldrich), which is an anti-fade mounting medium. Labeled neurons were visualized using a confocal microscope (Zeiss SLM 510 Meta).

The specificity of the immunostaining protocols above was tested by incubating cultured slices without the primary antibody. No immunostaining was observed in these slices.

### Statistics

Averages are expressed as mean ± SEM. Differences between groups were evaluated for the burst rates and the total activity using the *T*-test and for the number of active channels using the Chi-square test. Significance was accepted when *p* < 0.05.

### Chemicals and Drug Application

All chemicals were dissolved in extracellular solution at the final concentration. For drug application, the bath solution was exchanged twice with the drug-containing solution using a syringe, resulting in a final exchange of the bath solution by about 90–95%. For prolonged drug application (>15 min) the bath solution was exchanged with a drug-containing solution several times. The following agents were used: CNQX (6-cyano-7-nitroquinoxaline-2,3-dione), D-APV (D-(2R)-amino-5-phosphonovaleric acid), riluzole (2-amino-6-(trifluoromethoxy)benzothiazole), flufenamic acid (FFA), 9-phenanthrol, gadolinium chloride, clemizole hydrochloride and ML204 (4-Methyl-2-(1piperidinyl)-quinoline; all Sigma); TTA-P2 (3,5-dichloro-N-[1-(2,2-dimethyl-tetrahydropyran-4-ylmethyl)-4-fluoro-piperidin-4-ylmethyl]-benzamide): Alomone Labs; gabazine (2-(3-Carboxypropyl)-3-amino-6-(4methoxyphenyl)pyridazinium bromide: Abcam).

## Results

### Spontaneous Activity in Longitudinal Slice Cultures

To study circuits from the ventral spinal cord *in vitro* we cultured longitudinal slices of lumbar ventral rat spinal cord (see Figure 1B). Similar to what we have described before for transverse slice cultures, all of the longitudinal slice cultures showed spontaneous activity (mean activity: 15.8 ± 14.7 events/s/channel) that was organized in network bursts (see Figure 1A). Such bursts consisted of periods with high rates of simultaneous multi-unit activity at many electrodes that were interrupted by periods of low network activity. Bursts appeared at a rate of about 1–20 per minute (mean rate: 5.8 ± 5.6/min, *n* = 27) and lasted for about 100 ms up to several tens of seconds (mean burst duration: 10.4 ± 15.4 s, *n* = 27). Activity during burst was spread over the whole slices with some preference for the edges (see Figure 1B). Most of the bursts contained intra-burst oscillations (see Figure 1A) with frequencies of 3–20 Hz (mean initial frequency: 12 ± 6.2 Hz, *n* = 24, that usually became slower towards the end of the bursts). Bursts usually started from several preferential sites that we called burst sources (mean number of burst sources per culture: 4.9 ± 2.4, *n* = 25). These sites were randomly spread over the whole area of the slices (see Figure 1C).

As in the circuits of transversal slice and of dissociated neurons (Streit et al., 2001; Tscherter et al., 2001), we recorded intrinsic activity in the cultures after synaptic disconnection of the networks in the presence of blockers of excitatory synaptic transmission. When the glutamatergic synaptic transmission was blocked with the AMPA receptor antagonist CNQX (10 μM), the bursts disappeared and were replaced by asynchronous activity at low rates (1.7 ± 1.1 events/s/channel, *n* = 13) in a fraction of channels (in 34.1 ± 21.9% of the active channels, *n* = 13, see Figures 1D–F). Again, the sites of the channels with such spontaneous intrinsic activity were spread over the slices in different cultures without obvious preferential sites except a slight preference for the rims of the slices (see Figure 1E), similar to the total activity and the burst sources.

Together these findings suggest that the spontaneous activity in cultures of longitudinal ventral horn slices is based on similar mechanisms as previously proposed for spinal cord networks in cultures from transverse slices and dissociated spinal cord neurons (Tscherter et al., 2001; Darbon et al., 2002; Yvon et al., 2005; Czarnecki et al., 2008): irregular spontaneous intrinsic activity in a fraction of neurons cause bursts and oscillations through recurrent excitation in synaptically coupled networks.

### Bursting and Intrinsic Activity Are Based on I_NaP_ and I_CAN_

Using pharmacological ion channel blockers, we next investigated which inward currents may be involved in the spontaneous intrinsic activity. We have previously found that persistent sodium currents (I_NaP_) are involved in the generation of intrinsic firing and spontaneous bursting in spinal cord networks in culture (Darbon et al., 2004; Czarnecki et al., 2008). We, therefore, tested the effect of the I_NaP_ blocker riluzole at high doses of  20 μM on intrinsic and bursting activity. At such doses, riluzole has been shown to completely block I_NaP_ with minor effects on transient sodium currents and thus on evoked single spikes (Czarnecki et al., 2009).

**Figure 3:**
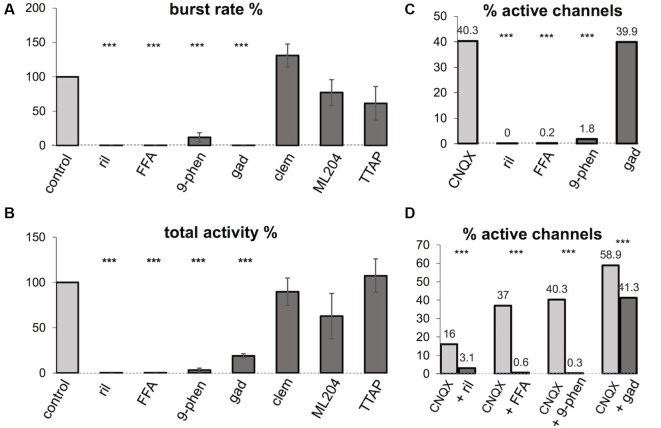
Effects of various ion channel antagonists on spontaneous burst rate and intrinsic activity. ril: 20 μM riluzole; FFA: 100 μM flufenamic acid; 9-phen: 100 μM 9-phenanthrol; gad: 30 μM gadolinium chloride; clem: 3 μM clemizole hydrochloride; ML204: 10 μM ML204; TTAP: 1 μM TTA-P2; CNQX: 10 μM CNQX. **(A,B)** Normalized burst rate and total activity after exposure to the antagonist for 30–60 min in percent of the control value (± SEM). ***Significant difference to control (*p* < 0.001, *n* = 5–21 experiments per antagonist, *T*-test). **(C)** Number of active channels after exposure to the antagonist for 30–60 min in percent of the control values (number of active channels during bursting activity). ***Significant difference to CNQX (*p* < 0.001, *n* = 5–21 experiments per antagonist, Chi-square test). **(D)** Effects of channel antagonists on the number of active channels in the presence of CNQX in percent of the control values during bursting activity. ***Significant difference to CNQX (*p* < 0.001, *n* = 4–10 experiments per antagonist, Chi-square test).

Riluzole decreased the rate and duration of bursts leading to a complete block of bursting within 15–45 min (see Figures 2A, 3A,B; *p* < 0.001, *n* = 7, *T*-test). In contrast to CNQX, no spontaneous intrinsic activity persisted after cessation of bursting (0/368 active channels in seven cultures vs. 243/710 active channels with CNQX in 13 cultures, see Figures 2A, 3C; *p* < 0.001, Chi-square test). In the presence of CNQX, riluzole decreased the number of active channels from 31 to 6/193 channels in four cultures (see Figure 3D; *p* < 0.001, Chi-square test).

Next, we tested the contribution of I_CAN_ to intrinsic activity and spontaneous bursting. To our surprise, we found that the I_CAN_ blocker FFA (100 μM) had practically the same effects on spontaneous activity as riluzole. It reduced burst rate and duration up to a complete cessation of spontaneous bursting (see Figures 2B, 3A,B; *p* < 0.001, *n* = 8, *T*-test) without leaving spontaneous intrinsic activity after 20–30 min (3/670 active channels in 10 cultures, see Figures 2B, 3C; *p* < 0.001, Chi-square test). Also, FFA suppressed intrinsic activity in the presence of CNQX (from 179 to 3/484 active channels in 10 cultures, see Figure 3D, *p* < 0.001, Chi-square test).

Since FFA interferes with a variety of ion channels (Guinamard et al., 2013) we tested the more specific I_CAN_ blocker 9-phenanthrol for its effects on spontaneous bursting and intrinsic firing in conventional slice cultures. 9-phenanthrol is reported to specifically inhibit the TRPM4 ion channel (Guinamard et al., 2014). Like FFA, 9-phenanthrol (100 μM) completely suppressed spontaneous bursting (see Figures 2C, 3A,B; *n* = 8, *p* < 0.001, *T*-test) and asynchronous activity within 30–60 min (to 2/112 active channels in 13 experiments, see Figures 2C, 3C; *p* < 0.001, Chi-square test). In the presence of CNQX, 9-phenanthrol reduced the active channels from 147 to 1/365 in 10 experiments (see Figure 3D; *p* < 0.001, Chi-square test).

To investigate the contribution of other channels that are possibly involved in I_CAN_, we further tested the effects of gadolinium chloride, an unspecific blocker of I_CAN_ and stretch-activated ion channels (Adding et al., 2001), and of the TRPC blockers clemizole chloride (TRPC5) and ML204 (TRPC4). Gadolinium (30 μM) fully suppressed bursting within 30 min (see Figures 2D, 3A,B; *n* = 5, *p* < 0.001, *T*-test), but did not suppress the asynchronous intrinsic activity compared to CNQX (118/296 active channels in eight experiments, see Figures 2D, 3C, *p* = 0.89, chi-square test). In the presence of CNQX, it slightly reduced the active channels from 96 to 67/163 in four experiments (see Figure 3D; *p* < 0.005, chi-square test) but did not fully suppress intrinsic activity. This effect was therefore clearly distinct from the effects of FFA and 9-phenanthrol.

Clemizole (3 μM) and ML204 (10 μM) had no effect on burst rate (see Figure 3A; *p* = 0.27 and 0.06, respectively, *n* = 5, *T*-test) and total activity (see Figure 3B; *p* = 0.56 and 0.08, respectively, *n* = 5, *T*-test) within 50 min.

Taken together, these results suggest that I_NaP_ through sodium channels and I_CAN_ through TRPM4 channels are the main contributors that control spontaneous intrinsic firing of spinal cord neurons in culture and network bursting based on this intrinsic activity. The finding that blocking one current suppresses intrinsic activity at almost all electrodes in the network excludes the hypothesis, that two different populations of neurons with distinct intrinsic firing mechanisms are present. More likely, spontaneous intrinsic firing is based on the cooperation of I_NaP_ and I_CAN_ in individual neurons. Since I_NaP_ is activated by depolarization in the range of −60 to −30 mV while I_CAN_ is activated by rising intracellular Ca^2+^, we hypothesized that low-voltage-activated calcium channels (T-type calcium channels) may act as a link for the cooperation of the two currents for spike generation. We, therefore, investigated whether the specific T-type calcium channel blocker TTA-P2 has an effect on bursting and total spontaneous activity in our cultures. We found no effect of 1 μM TTA-P2 on burst rate (see Figure 3A; *p* = 0.07, *n* = 5, *T*-test) or total activity (see Figure 3B; *p* = 0.28, *n* = 5, *T*-test).

### Involvement of I_CAN_ and I_NaP_ in Bursting Activity Induced by Electrical Stimulation

**Figure 4:**
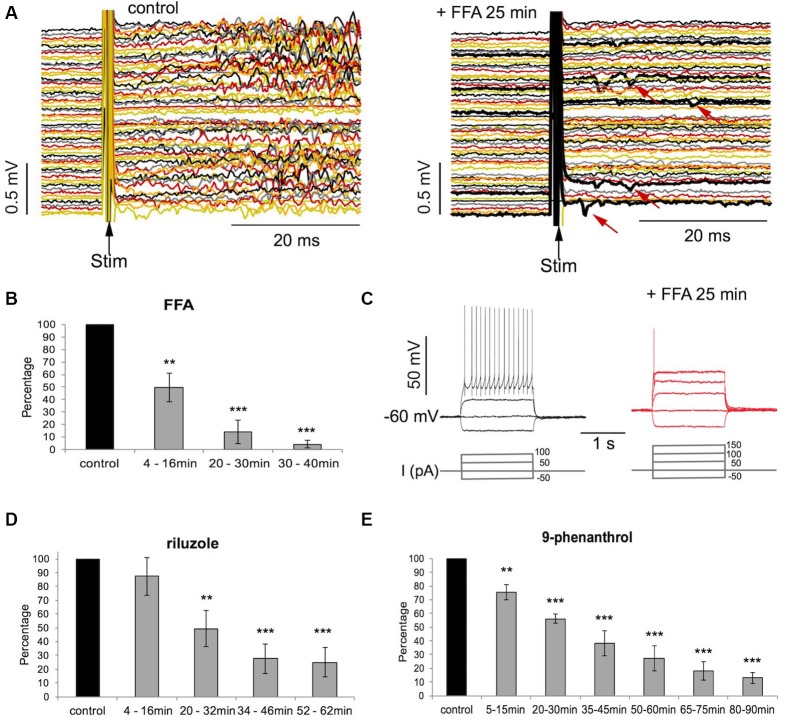
Evoked activity is suppressed by FFA, riluzole, and 9-phenanthrol. **(A)** MEA recordings of activity in 60 channels evoked by electrical stimulation before and after exposure to FFA (100 μM). Note the few spikes (arrows) evoked by stimulation in FFA. **(B)** Decrease of evoked activity (in % of control) with time of FFA exposure (*n* = 8, **p < 0.01; ***p < 0.001, *T*-test). **(C)** Intracellular patch-clamp recordings from one neuron during current injection. Note that FFA evoked repetitive firing is reduced to a single spike and activation threshold is increased by one current step (50 pA). **(D,E)** Decrease of evoked activity (in % of control) with time of exposure to riluzole (*n* = 7, **p < 0.01; ***p < 0.001, *T*-test) and 9-phenanthrol (*n* = 7, **p < 0.01; ***p < 0.001, *T*-test).

We have previously shown that riluzole at 10–20 μM suppresses repetitive firing during sustained depolarization in individual neurons and the generation of network bursts by extracellular electrical stimulation (Darbon et al., 2004). We, therefore, tested whether FFA has similar effects as riluzole in suppressing bursting activity that is evoked by electrical stimulation. We found that FFA suppressed stimulus-evoked bursts of activity within 20–30 min while leaving few individual responses to the stimuli intact (see Figure 4A; *p* < 0.001, *n* = 8; *T*-test), thereby reducing the stimulus-evoked network bursting activity to less than 10% of control values (see Figure 4B). Riluzole and 9-phenanthrol also strongly reduced stimulus-induced network bursting activity to about 20% of control (see Figures 4D,E; *p* < 0.001, *n* = 7, *T*-test).

To investigate the effect of FFA on the repetitive firing of neurons during sustained depolarization we injected depolarizing and hyperpolarizing current pulses (2 s long, 50–300 pA in 50 pA steps) into four neurons using whole-cell patch-clamp. We found that in all neurons injection of depolarizing current pulses at spiking threshold (100–200 pA) evoked repetitive firing at an average number of 14 ± 3.4 spikes during the 2 s pulses (see Figure 4C). FFA (100 μM) caused a progressive suppression of repetitive firing leading to single spikes after 25 min of drug application at threshold currents (100–200 pA; see Figure 4C). The rheobase current in these 4 cells increased from 100–150 pA to 150–200 pA (one step) with FFA. During the injection of the strongest current pulses of 200–300 pA, the average number of spikes in the four cells was suppressed from 54 ± 43 to 1.5 ± 0.5 (1–2) per 2 s with FFA. The input resistance did not change with FFA in these four neurons (214 ± 77.2 MΩ in FFA vs. 176 ± 33.5 MΩ, *p* = 0.299, *T*-test).

Together these findings suggest that both I_CAN_ and I_NaP_ also contribute to repetitive firing during sustained depolarization of neurons and thereby to the size and duration of network bursts.

### Do Hb9 Interneurons Have a Role in Burst Generation?

**Figure 5:**
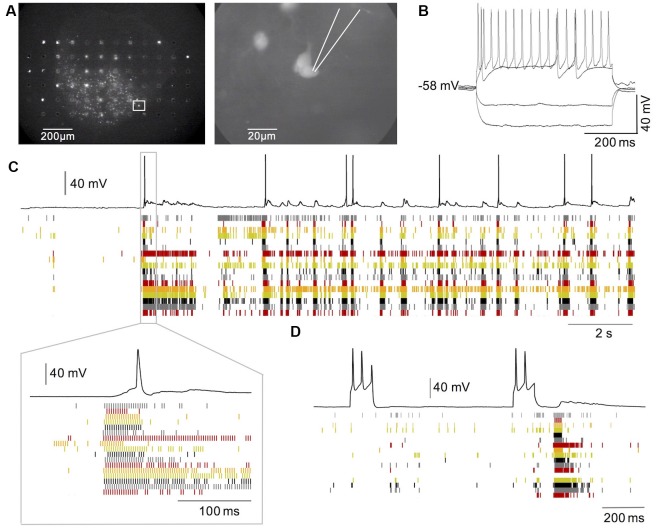
Network-driven activity in GFP-labelled HB9 interneurons. **(A)** GFP labeled neurons in a spinal cord slice culture from HB9-GFP mice on MEA. **(B)** Repetitive firing induced in such a neuron by current injection. **(C)** Intracellular recording from one GFP neuron (black trace) in combination with MEA recordings (raster plot). Note that synaptic and spiking activity in the neuron is correlated to the network activity and follows network bursts with a delay. **(D)** Combined single neuron and network recording during activation of the neuron through current injection. Note that repetitive firing in the neuron cannot evoke network bursts.

It has been proposed and debated whether Hb9 excitatory interneurons have a critical role in the generation of rhythmic activity in spinal cord circuits (Ziskind-Conhaim et al., 2010; Dougherty et al., 2013; Ljunggren et al., 2014; Caldeira et al., 2017) and whether persistent sodium currents play a crucial role in such rhythm generation (Tazerart et al., 2008; Ziskind-Conhaim et al., 2008). We, therefore, investigated whether Hb9 interneurons may have a critical role in the initiation of spontaneous bursts in spinal cord slice cultures. Since our low-density MEA recordings from slice cultures did not allow us to directly assign signals from MEA electrodes to immunohistochemically identified neurons, we combined MEA recordings with single-cell recordings from GFP labeled neurons in transverse slice cultures from the spinal cord of e13 Hb9-GFP mouse embryos. As shown in Figures 5C,D, spontaneous bursts of network activity that are similar to those described in cultures from rat slices also appear in cultures of embryonic mouse spinal cord slices (Avossa et al., 2003; Furlan et al., 2007). GFP positive neurons were visualized using fluorescence microscopy (see Figure 5A) and single-cell recordings were made using the whole-cell patch-clamp method. To exclude putative motoneurons from this analysis, we stained the cultures for ChAT after the experiments. By comparing the pictures from the recorded cells to the ChAT stainings, of the same cultures on the MEAs we selected 31 GFP-labeled neurons that were not stained positive for ChAT in 14 cultures for this analysis. These neurons had a mean resting membrane potential of −52.9 ± 5.4 mV (±SD). During the injection of depolarizing current pulses, 28 of 31 showed repetitive firing as shown in Figure 5B. All of these neurons showed synaptic potentials that were correlated with the network bursts, but only 18 of them showed spontaneous spiking during network bursts as shown in Figure 5C. Neurons with spontaneous spikes were significantly more depolarized than those without spontaneous spikes (−50.3 ± 5.1 vs. −56.4 ± 3.7 mV; *n* = 18 vs.13; *p* < 0.001). The spikes always rode on synaptic potentials with a delayed onset relative to the start of the network burst as shown in Figure 5C. Trains of repetitive spikes that were evoked by current injection in individual Hb9 interneurons never evoked network bursts (*n* = 28 neurons, see Figure 5D). Spiking activity in these neurons was thus driven by the network bursts but did not initiate them.

## Discussion

The main finding of this study is that the I_CAN_ blockers FFA and 9-phenanthrol, similar to the I_NaP_ blocker riluzole, completely block intrinsic activity and spontaneous bursting in spinal cord networks and strongly reduce stimulus-evoked network responses. The finding that both riluzole and FFA suppress spontaneous intrinsic activity in disconnected networks at almost all electrodes is not compatible with the hypothesis that I_NaP_ and I_CAN_ contribute to the generation of intrinsic spiking in two distinct populations of neurons (Del Negro et al., 2005). More likely it suggests that I_CAN_ and I_NaP_ cooperate in individual neurons to support intrinsic and repetitive firing.

### Mechanisms of Spontaneous Network Bursting in Spinal Cord Circuits in Culture

In the present experiments, we found spontaneous bursts of activity, often with intraburst oscillations in cultures of longitudinal ventral slices of the lumbar spinal cord of E14 rat embryos cut in the frontal plane. As in transverse slices, we found both short and long bursts with synchronous oscillations in the whole slice. These patterns of activity are similar to those previously reported in cultures of transverse spinal cord slices (Ballerini et al., 1999; Tscherter et al., 2001; Czarnecki et al., 2008) and also resemble the patterns called superbursts that appear in some cultures of dissociated cortical neurons (Wagenaar et al., 2006). The neuronal circuits in spinal cord cultures from both transverse and ventral longitudinal slices are therefore capable to generate synchronous bursting activity while lacking other aspects of organotypic pattern generation in isolated spinal cord preparations or *in vivo* like alternating patterns between the left and the right side of the spinal cord (Kiehn, 2016). As previously reported for networks in transverse slices (Czarnecki et al., 2008), such spontaneous bursting patterns involve recurrent network excitation through glutamatergic synaptic circuits since the block of glutamatergic transmission suppressed the bursting and left irregular asynchronous spontaneous activity in roughly 30% of the channels (see Figure 1D). We have previously shown in cultures of dissociated spinal neurons and transverse slices that such asynchronous activity represents spontaneous intrinsic spiking in part of the neurons (Darbon et al., 2002). Such intrinsic activity can be blocked by riluzole and thus depends on I_NaP_ (Darbon et al., 2004; Czarnecki et al., 2008).

I_NaP_ has also been shown to be involved in the generation of intrinsic spiking and in pacemaker activities in CPG networks of the spinal cord (Tazerart et al., 2007, 2008; Ziskind-Conhaim et al., 2010), the brainstem (Pace et al., 2007b) and the cortex (Le Bon-Jego and Yuste, 2007).

### Contributions of I_NaP_ and I_CAN_ in Circuits of the Ventral Spinal Cord

In the pre-Bötzinger nucleus of the brain stem, inspiratory pattern generators were proposed to operate with two types of pacemaker neurons with different mechanisms that drive intrinsic firing. One is based on I_NaP_, the other on I_CAN_ (Del Negro et al., 2005). However, it is not clear to what extent I_CAN_ activates cells from resting potential or whether it needs additional currents for depolarization since I_CAN_ is known to be involved in the generation of plateau potentials in motoneurons and neocortical neurons (Schiller, 2004; Del Negro et al., 2010). In the spinal cord, there is so far no evidence for the involvement of I_CAN_ in pattern generator networks. In the lamprey, some effects of FFA are reported but attributed as rather unspecific effects than being indicative of a contribution of I_CAN_ (Wang et al., 2006). Only in pain circuits of the dorsal spinal cord, pacemaker neurons are reported to potentially depend both on I_NaP_ and I_CAN_ (Li and Baccei, 2011). Our finding that the I_CAN_ blockers FFA and 9-phenanthrol completely suppress spontaneous bursting and intrinsic firing in cultures of longitudinal slices from ventral spinal cord show, that I_CAN_ also critically contributes to intrinsic firing and spontaneous rhythm generation in circuits of the ventral mammalian spinal cord. Furthermore, since blockers of I_NaP_ and I_CAN_ both completely block intrinsic firing at all electrodes, a distinct population of neurons with different mechanisms of intrinsic spiking are unlikely. Instead, these findings are more compatible with the hypothesis that both I_CAN_ and I_NaP_ contribute to intrinsic spiking in individual neurons and thus to rhythm generation in the network. This view is similar to the concept of a group pacemaker that was recently proposed for rhythm generation in the pre-Bötzinger complex (Del Negro et al., 2018).

We have previously shown that at the concentration used for this study (20 μM), riluzole suppresses repetitive spiking during current injection but leaves the initial spike intact (Darbon et al., 2004), showing that the suppression of intrinsic spiking is not simply due to the suppression of spike generation in general. Here, we show the same effects for the I_CAN_ blocker FFA: repetitive firing in individual neurons during injection of depolarizing current pulses is suppressed and reduced to one or two spikes by FFA. Also, both FFA and 9-phenanthrol reduce stimulus-induced network bursts to about 20% of control. Together these results suggest that I_CAN_ and I_NaP_ contribute to both intrinsic firing and repetitive firing during sustained depolarization.

The identity of intrinsically firing neurons in culture is not clear. Among others, excitatory Hb9 interneurons have been discussed as pacemaker neurons in rodent CPGs (Ziskind-Conhaim et al., 2008). In line with this hypothesis, we found a spontaneous activity that is correlated to network bursts in the majority of HB9 interneurons in HB9 GFP mice. However, for none of these neurons could activation of the neuron evoke network bursts. These findings argue against a prominent role of HB9 interneurons as intrinsically active cells that can initiate spontaneous network bursts in spinal cord cultures. It does however not exclude that these neurons may represent an important pacemaker under *in vivo* conditions or in more acute preparations (Ziskind-Conhaim et al., 2010; Caldeira et al., 2017).

### Possible Involvement of TRPM4 Channels

Our conclusion critically depends on the assumption that the effects of FFA are due to the block of I_CAN_. In neurons, the effective dose of FFA to block I_CAN_ is reported to be around 100 μM (Pace et al., 2007a; Li and Baccei, 2011; Guinamard et al., 2013; Tsuruyama et al., 2013). At such doses, however, it is highly unspecific and has many other effects in addition to blocking I_CAN_. FFA was initially developed as a non-steroidal anti-inflammatory drug that inhibits the cyclo-oxygenases. This compound turned out to have a variety of effects on receptors and ion channels of the central nervous system (for review see Guinamard et al., 2013). At the concentration used in this study, it blocks several types of TRP channels (mainly belonging to the families of TRPC, TRPM and TRPV), chloride channels (including GABA A channels), Connexins, L-type Ca^2+^ channels, NMDA channels and nicotinic channels. Furthermore, at higher doses than used in this study (200 μM), FFA can interfere with sodium channel inactivation and thereby contribute to the suppression of repetitive firing (Yau et al., 2010) and it can even activate some channels like TRPA and nicotinic channels. Nevertheless, we believe that the effects reported here are mainly due to a block of TRPM4 channels since 9-phenanthrol, a specific blocker of the TRPM4 channel (Guinamard et al., 2014), could fully reproduce the effects of FFA on intrinsic activity and on spontaneous and evoked bursting. However, also 9-phenanthrol at doses used in this study (100 μM) can have unspecific effects (Guinamard et al., 2014). Therefore, we tested other inhibitors of unspecific cation currents in terms of their effectiveness to block intrinsic activity and network bursting. In contrast to 9-phenanthrol, these blockers only reproduced some of the effects of FFA on rhythmic activity like gadolinium chloride, an unspecific blocker of I_CAN_ that had its major effects on stretch-activated ion channels (Adding et al., 2001), or they had no effects like the TRPC4 antagonist ML204 (Miller et al., 2011) or the TRPC5 antagonist clemizole (Richter et al., 2014). TRPC4/5 channels are reported to contribute to seizure generation in hippocampal circuits (Phelan et al., 2013; Zheng, 2017). We have previously shown that block of connexins or nicotinic channels in transverse slice cultures reduces spontaneous activity, but never completely blocked spontaneous bursting and intrinsic activity (Magloire and Streit, 2009). Also, the NMDA blocker APV and the GABA A blockers bicuculline and picrotoxin increased spontaneous activity in spinal cord slice cultures (Czarnecki et al., 2008). In summary, TRPM4 channels are the most likely structures underlying I_CAN_ in our experiments. TRPM4 channels are expressed in neurons and axons of mouse and human spinal cord (Schattling et al., 2012) and are involved in the output of the breathing CPG in mice (Koizumi et al., 2018; Picardo et al., 2019). Nevertheless, we cannot exclude the involvement of other unspecific cation channels of the TRPM, TRPC or TRPV families that are inhibited by FFA. Some of these channels like TRPC3/7 (Ben-Mabrouk and Tryba, 2010; Koizumi et al., 2018; Picardo et al., 2019), TRPM2/4 (Mrejeru et al., 2011) or TRPV2 (Bouhadfane et al., 2013) have been shown to play a role in rhythm or pattern generation in mammalian neuronal circuits.

The mechanisms involved in the block of the TRPM4 channels by FFA and 9-phenanthrol are unknown. It, therefore, remains an open question why these effects develop so slowly. The effects of CNQX rapidly occurred within seconds. Therefore, the slow establishment of effects cannot be attributed to the mode of application that was the same for all drugs. Both FFA and 9-phenanthrol, as well as riluzole, are lipophilic substances (Guinamard et al., 2013, 2014). It has been shown for several lipophilic anesthetics like propofol (Gredell et al., 2004) as well as etomidate and thiopental (Voss et al., 2013) that they show slow diffusion into mammalian cortical slices causing equilibrium times for drug distribution and effectiveness in the range of hours. Although the diffusion coefficients of FFA, 9-phenanthrol or riluzole are not known, they may be low enough to cause slow diffusion and thus the slow establishment of effects even in the relatively thin slices present in the spinal cord cultures.

We can only speculate about the mechanism of cooperation between TRPM4 and sodium channels at the resting membrane potential to intrinsically activate the neurons. A possible link would be T-type calcium channels that are activated at voltages between the resting membrane and the threshold potential and could thus be opened by depolarization through I_NaP_ and activate I_CAN_ by increasing intracellular Ca^2^. T-type calcium channels have been proposed to be implicated in rhythm generation in mouse spinal cord (Anderson et al., 2012). However, in the present study, the specific T-type blocker TTA-P2 (Choe et al., 2011) did not affect spontaneous bursting and intrinsic spiking. This finding excludes a critical role of T-type calcium channels in the cooperation between I_CAN_ and I_NaP_. Another possibility is that the neurons are depolarized into the activation range of I_NaP_ through TRPM4-induced fluctuations in membrane potential induced by intracellular calcium. Spontaneous intracellular calcium fluctuations have indeed been reported in spinal cord slice cultures (Fabbro et al., 2007). This hypothesis needs further investigation in future experiments.

In summary, we propose that I_NaP_ through sodium channels and I_CAN_ through putative TRPM4 channels jointly contribute to the generation of intrinsic and repetitive firing in intrinsically active neurons and thus to the generation of network bursting in the spinal cord circuits in culture.

## Data Availability Statement

The raw data supporting the conclusions of this article will be made available by the authors, without undue reservation, to any qualified researcher.

## Ethics Statement

The use of animals for the preparation of slice cultures for this study was reviewed and approved by Swiss local authorities: Amt für Landwirtschaft und Natur des Kantons Bern, Veterinärdienst, Sekretariat Tierversuche, approval Nr. BE 52/11 and BE 35/14.

## Author Contributions

JS designed and coordinated the research. SB, AT, MH and JS performed research and analyzed data. JS wrote a first draft of the manuscript. All authors contributed to and approved the final manuscript.

## Conflict of Interest

The authors declare that the research was conducted in the absence of any commercial or financial relationships that could be construed as a potential conflict of interest.
